# Effects of high‐protein milk powder, linseed paste, and grape molasses levels on physiochemical, rheological, and sensory attributes of linseed spread

**DOI:** 10.1002/fsn3.3309

**Published:** 2023-03-13

**Authors:** Siamak Rahbari, Hamid Tavakolipour, Ahmad Kalbasi‐Ashtari

**Affiliations:** ^1^ Department of Food Science and Technology, Tehran North Branch Islamic Azad University Tehran Iran; ^2^ Department of Food Science and Technology, Sabzevar Branch Islamic Azad University Sabzevar Iran; ^3^ Biological and Agricultural Engineering Department Texas A&M University College Station Texas USA

**Keywords:** sensory properties, high‐protein milk powder, linseed spread, mathematical modeling, persian grape molasses, texture

## Abstract

Separate levels of roasted linseed paste (RLP) (15, 22.5, and 30 g), Persian grape molasses (PGM) (40, 50, and 60 g), and high‐protein milk powder (HPMP) (3.75, 6.5, and 9.25 g) were ground and mixed in a ball mill (3 h at 45°C) to make samples of linseed spread (LS). After applying response surface methodology and central composite design, the optimized LS was obtained with 22.5 g RLP, 50 g PGM, 6.5 g HPMP, fine particle sizes (<30 μm), and 92% of the highest acceptable sensory scores (ASS). The mathematical model of ASS had good fitness (*R* ^2^ > 95%) with the ingredient's levels of LS samples. While the PV, aw, and acidity of optimized LS did not change even after 90‐day storage at 4°C, it showed viscoelastic properties and very low stickiness (0.2–0.4 mJ). Additionally, the hardness, adhesiveness, cohesiveness, springiness, gumminess, and chewiness of optimized LS, respectively, decreased by 50, 25, 3, 8, 55, and 63% when its temperature increased from 4 to 25°C.

## Graphical abstract

This project we studied the possibility of making nutritional spread (similar to Nutella) from the linseed, Persian grape molasses, and high protein milk powder for the first time. After applying response surface methodology and central composite design, the optimized‐LS obtained with 22.5g RLP, 50g PGM, 6.5g HPMP, fine particle sizes (< 30µm), and 92% of the highest acceptable sensory scores (ASS).

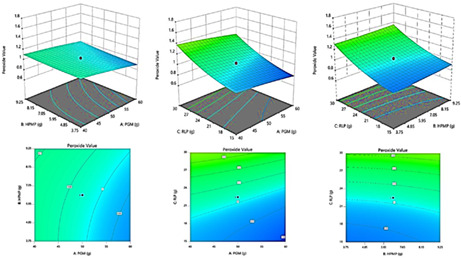

## 1 INTRODUCTION

Although linseeds and flaxseeds have different botanical specifications, they are nutritionally like each other. While people in the UK and Australia distinguish between linseed and flax, in the United States and Canada, they refer to both as flax (Food Lovers Market, 2022). The world linseed production reached 3.4 million tons in 2020; however, its Asia portion was 1.5 million (~50%) (Knoema Corporation, 2022). The linseed (*Lallemantia iberica* L) received considerable attention because of its considerable polyunsaturated fatty acids (PUFA), notably alpha‐linolenic acid, omega‐3 fatty acid, and conjugated linoleic acid (CLA), which are essential and healthy compounds for human brain activities (Heuzé et al., 2015). Additionally, the linseed cake or meal resulting from cold pressing of its seeds has 40% protein, 6% minerals (ash), and 12% oil (Mueller et al., 2010); and it is the most effective material for roasting and making linseed paste with interesting sensory attributes (Farag et al., 2021). The insoluble dietary fiber component of linseed (~7%) is effective in constipation relief and colon health and may even protect against colon cancer (Condori et al., 2020).

Different milk spreads are produced by using ball milling system most of the times to reduce the particle size of diverse ingredients (different nuts, milk and/or cocoa powder, and cocoa butter) (Amevor et al., 2018).

Many people like the excellent taste of milk and nut spreads because they are melted on their tongue due to their fine particles. Additionally, the gradual breaking of their mixed ingredients removes the undesirable flavors of used ingredients and develops a pleasant aroma in the final spread. In other words, the heterogeneous, flaky, and relatively dried paste of each ingredient in LS product is transformed into free‐flowing solid and fine particles dispersed in a dominant fat phase of spreads (Acan et al., 2021). The inclusion of 10%–20% milk powder in any kind of nut spread affects its physiochemical, rheological, and sensory characteristics (Monteiro et al., 2018). Furthermore, the method of milk drying (roller or spray dehydration) for its conversion to powder has effects on sensory attributes of final spread when it is used as an ingredient (Coutinho et al., 2019; Crawford & Running, 2020).

Since there is a trend to reduce crystal sugar (as a sweetening agent) in most of the food products, it was our interest to substitute crystal sugar with the native and local Persian grape molasses (PGM). While PGM has more than 50% sugar (mainly glucose and fructose) and depends on its Brix, it has considerable minerals, organic acid, and bioactive compounds that are made from grape or raisin (Azizi‐Tabrizzad, 2020). Since PGM has interesting rheological properties and organoleptic (texture, color, and flavor) specifications, it will enhance the quality aspects of different products, if it is used properly as an ingredient in food products (Azizi‐Tabrizzad, 2020).

Our objectives were to study the effects of three ingredients (roasted linseed paste (RLP), high‐protein milk powder (HPMP), and Persian grape molasses (PGM)) on physiochemical, rheological, and sensory attributes of linseed spreads (LS). The quality indicators (peroxide values, water activity, and acidity) of LS were evaluated for 3‐month storage at 4°C. Additionally, statistical methods including response surface method and central composite design were applied to find out the optimized levels of RLP, HPMP, and PGM. It was our hypothesis to make a highly nutritional and novel LS with high sensory scores and minimum deteriorating indexes.

## 2 MATERIALS AND METHODS

### 2.1 Materials

The linseed, seedless grape, null flour of wheat, skim milk powder, and liquid lecithin were procured from local markets and factories established in different parts of Iran. The null flour was obtained by grinding the core part of endosperm available in semi‐soft or hard wheat. It contained fully even texture and soft graining (fine particles) as well as high protein quality. Furthermore, the color of this kind of wheat flour was clear, without any bran or spots. The permissible ranges of ash, gluten, and protein in this flour are 0.38%–0.50%, 20%–27%, and 7%–10%, respectively. Milk protein (with 85% concentration) was prepared from Alinda Company (Greece), and the needed chemical compounds were purchased from Merck Corporation (Germany).

### 2.2 Preparation methods, measuring specifications, and modeling

#### 2.2.1 Preparation of Persian grape molasses (PGM) and high‐protein milk powder (HPMP)

The stems and clusters of prepared seedless grapes were removed from their clusters and washed. Then, its juice was prepared by pressing in a juice extractor and blended with bentonite powder (3 g per 100 mL of grape juice) to amend its acidity. Then, the modified grape juice was mixed with clarifying local earth, heated for concentration (until its brix became >30%), and filtered (Tavakolipour et al., 2020). Later, the semiconcentrated grape juice was mixed with enough calcium carbonate to increase its pH to ~ 5.6. Next, the subsequent product was filtered and cooked under a vacuum to make Persian grape molasses (PGM) with brix of ~70% (Tavakolipour et al., 2020). After measuring the ash (=1.75%), pH (=5.6), and acidity (=0.162%) of the resulting PGM, it was packed and stored in a sterile container for future use.

The high‐protein milk powder (HPMP) was made by mixing skim milk powder and milk protein concentrate (1:1) and the ready uniform powder was packed in sterile plastic bags until further usage.

#### 2.2.2 Preparation and processing of linseed spread (LS)

Linseed paste with three levels (15, 22.5, and 30 g) was made by roasting its seeds at 121°C for 1 min followed by its uniform mixing with 1% lecithin (as emulsifier) at 45°C for 10 min (Turner & McNiven, 2011). Then, the null flour of wheat was roasted for 140 s at 180°C (Germishuys et al., 2020) and mixed separately with three levels (3.75, 6.5, and 9.25 g) of high‐protein milk powder (HPMP) at a rate of 2.5%. Later, three levels (40, 50, and 60 g) of PGM were added separately and evenly to the resulting mixture and the content of each level of ingredient was stirred for 10 min at 25°C to develop a liquor phase (Turner & McNiven, 2011). The mass of ultimate mixture was made to flow through a ball mill by using a recycling pump at a medium current of 1 kg/min. The LS was produced by using Sepehr ball mill (Sepehr machine, Iran) equipped with an agitator (600 RPM) containing 6‐mm‐diameter stainless‐steel balls using the technique of Bolenz et al. (2014). The particle size reduction and refining process were stabilized at 45°C and checked every 10 min during 3 h milling time. Whenever the temperature of mixed materials went above 45°C, the vessel was cooled indirectly with tap water. The ball milling was continued until the particle size reached ~30 μm. According to sensory scientists, the grinding process of solid and paste materials should be continued until they break into very fine particles (~30 um), which cannot be felt on the tongue (Amevor et al., 2018; Savitri et al., 2021). To prevent contamination, the prepared LS was shaped and wrapped in aluminum foil (Bolenz et al., 2014).

#### 2.2.3 Measuring chemical indexes (PV, aw, and acidity) and other properties of LS

Peroxide value (PV) and acidity (as a free oleic acid percentage) were evaluated by applying the AOCS method (AOCS, 1989). Water activity was determined at 25°C by using the instruction of Swiss aw Analyzer (Novasina AG, Switzerland). Total dietary fiber was determined by using the enzymatic gravimetric assay (based on the AOAC‐991.43 and AACC‐32.07.01 methods). Similarly, total protein was measured by using the AACC 46–30 method and a correction index of 5.95 for nitrogen‐to‐protein conversion. Fat content was measured by using a Soxhlet extractor (Velp, Italy) on 5 g of ground sample mixed with hexane as solvent. The ash content or mineral residue of each sample was determined by incineration of its total organic matter in a muffle furnace at 550°C, as described in the official AOAC international method (AACC, 2000; AOAC, 2000; Tobolková et al., 2021). Finally, pH was measured using a pH meter (IKA, Germany) which was calibrated before the test.

#### 2.2.4 Sensory evaluation

After selecting 20 members of taste panel (from the Department of Food Science at the University of Tehran), they were trained for sensory evaluation. The evaluators were seated in individual and separated kiosks equipped with air circulation under a bright light (recommended by ISO, 2007 ISO Standards No. 8589), and assessed 8–10 g of prepared LS samples (in nibble forms) 2 h after breakfast. They used a 5‐point hedonic scale to score different sensory attributes (appearance, flavor, mouth feel, melting, texture, and sweetness) of each LS sample, and each sample was tested three times. The final sensory acceptance score of samples is calculated by the average sum of three replicates of sensory attributes scores. Therefore, the maximum average sensory score, which could be gained for each sample was 100 points. Although this approach does not represent accurate customer perception, it strongly verified the required characteristics of a high‐quality LS product.

#### 2.2.5 Experimental design, model fitting, and variables optimization

The central composite design (CCD) system was used to study the effects of three independent variables of PGM, HPMP, and RLP on the peroxide value, a_w_, acidity, and sensory scores of the resulting LS samples. The design included 20 experiments, which consisted of six center points in a cube. The operating conditions were conducted at five levels coded as −2 (−α), −1, 0, +1, and + 2 (+α). Table 1 shows the actual and coded values of three independent variables.

**TABLE 1:** Actual and coded independent variables employed in the experimental design to find the specifications of optimized linseed spread after using response surface methodology.

Parameters	Coded value ^*^				
	−2 (−α)	−1 ‐	0 ‐	+1 ‐	+2 (+α)
PGM (Persian grape molasses) in (g)	30	40	50	60	70
HPMP (high‐protein milk powder)	1	3.75	6.5	9.25	12
RLP (roasted linseed paste)	7.5	15	22.5	30	37.5

^*^ The coded value or factor level of each variable used for making a matrix of central composite design applied in this study. Although only three levels of each ingredient were used to produce the LS with the highest sensory scores and permissible deteriorative indexes (PV, w_a_, and acidity), the response surface method (RSM) chose extra upper and lower levels (in addition to its three levels) for each ingredient to find the optimized formula for making linseed spread with the highest quality (sensory score) in spite of the chosen levels for each ingredient.

A second‐order polynomial model was employed to express the relationship between the independent variables and each dependent variable (α):

(1) $$\begin{array}{cc} \alpha =\beta_{0} & +\sum_{i=1}^{k}\beta_{i}x_{i}+\sum_{j=i+1}^{k}\beta_{j}x_{j}+\sum_{l=j+1}^{k}\beta_{l}x_{l}+\sum_{i=1}^{k}\sum_{j=i+1}^{k}\beta_{\mathrm{ij}}x_{i}x_{j}+\sum_{i=1}^{k}\sum_{l=j+1}^{k}\beta_{\mathrm{il}}x_{i}x_{l}\\ & +\sum_{j=i+1}^{k}\sum_{l=j+1}^{k}\beta_{\mathrm{jl}}x_{j}x_{l}+\sum_{i=1}^{k}\beta_{\mathrm{ii}}x_{i}^{2}+\sum_{j=i+1}^{k}\beta_{\mathrm{jj}}x_{j}^{2}+\sum_{l=j+1}^{k}\beta_{\mathrm{ll}}x_{l}^{2} \end{array}$$

Where *α* represents the response variable, β_0_ is the constant coefficient, x represents the independent variable (factor), *i, j,* and *l* are the number of independent variables, β_i_ β_j_ β_l_, β_ii_ β_jj_ β_ll_ and β_ij_ β_il_ β_jl_ are the linear, quadratic, and second‐order interaction for independent variables coefficients, respectively. The statistical software Design‐Expert®, version 11, was used for experimental design, analysis, graphing, and optimization.

Validation of the appropriate conditions was carried out in triplicate to generate the optimum model using Duncan's new multiple range test (MRT). The significance level used for this study was 95% (*p* < .05) for all the statistical analyses.

#### 2.2.6 Physical specifications (color values and texture profile analysis)

The Chroma Meter (Model D65, Minolta Co., Ltd., Japan) was used to measure color values including L (lightness), a (redness to greenness), and b (yellowness to blueness) of optimized LS samples in triplicate stored at 4°C for 0, 45 and 90 days. Additionally, it was calibrated using a Minolta calibration plate (Y = 94.75, x = 0.28; y = 0.44). The following equations were used to determine the CIELab properties including chroma, whitish (WI), yellowness (YI), and brownness (BI) indexes of samples based on color coordinates (Ekrami et al., 2019; Tobolková et al., 2021):

(2) $$\text{Chroma}=\sqrt{\;a^{2}+b^{2}}$$

(3) $$\mathrm{WI}=100-\sqrt{{\left(100-L\right)}^{2}+a^{2}+b^{2}}$$

(4) $$\;\mathrm{YI}=\frac{142.86\times b}{L}$$

(5) $$\;\mathrm{BI}=\frac{100\times \left(X-0.31\right)}{0.17}X=\frac{\left(a+\left(1.75\times L\right)\right)}{\left(5.645\times L\right)+a-\left(3.012\times b\right)}$$

The texture of each LS sample was measured with a Texture Analyzer (Stable Micro System Ltd., UK) and according to Nightingale et al. (Nightingale et al., 2011) method with some modifications. An aluminum cylinder probe with 50 mm diameter was attached to a 0.5 N compression load. Three speeds of 2.0 (as a pretest), 1.0 (as a test), and 2.0 mm/s (as a posttest) were used on the 1 cm^3^ of LS samples, and the instrument was adjusted to achieve 25% compression with 5‐s waiting periods. The texture curves were analyzed for hardness, cohesiveness, springiness, adhesiveness, gumminess, and chewiness. The attributes of hardness and chewiness were selected as essential response variables of final optimized LS.

#### 2.2.7 Rheological description

According to Fanari et al. (2020) method, the rheological properties of the prepared optimized LS were determined by using a stress/strain and temperature‐controlled rheometer (Anton Paar GmbH, Austria) equipped with parallel plates geometry (each one with 50 mm diameter). In the first, the gap between the two plates of rheometer was adjusted to 0.5 mm. After loading samples, its probe was descended and compressed the two plates with a gap of 2 mm. Then, viscosity measurement of optimized LS was started after waiting 2 min for temperature equilibration and product relaxation. The applied shear rate had a range between 1 and 100 s^−1^ radian frequency at 25°C. A power‐law model (Fanari et al., 2020) was used to show the dependency of the measured complex viscosity data on the frequency of LS.

(6) $$\sigma =K{\left(\gamma \right)}^{n}$$

Where σ is shear stress (Pa), K is the consistency coefficient (Pa·s^n^), γ is the shear rate (s^−1^), and n is the flow behavior index.

## 3 RESULT AND DISCUSSION

### 3.1 Chemical characterization of linseed spread including peroxide value (PV), water activity (aw), and acidity

The PV measurement is still one of the most important chemical indexes (CI) for determining how quickly the LS samples oxidize because of their oil and (to some extent) moisture content. When the three levels of PGM, HPMP, and RLP contents in LS formulation, respectively, changed from 50% to 60%, 3.75% to 6.5%, and 37.5% to 15%; the PV of resulting LS samples increased from 0.74 to 1.78 meq O_2_/kg after 90 days storage at 4°C. Similarly, aw and acidity increased, respectively, from 0.54 to 0.88 and 0.159 to 0.258 right after production.

**FIGURE 1:**
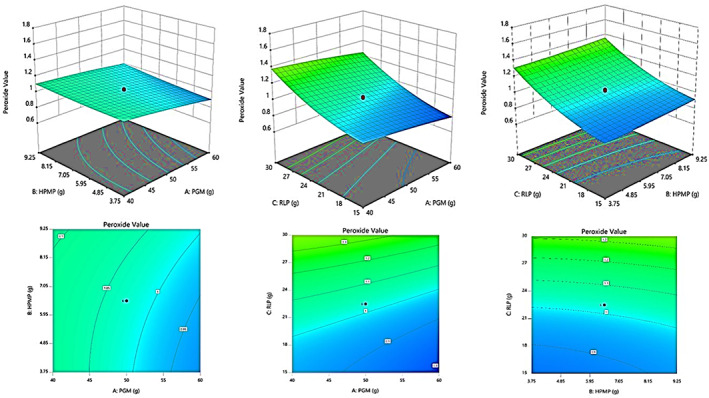
Response surface plots showing the interaction effects of independent variables (PGM, HPMP, and RLP each one with five levels) on the peroxide value (PV) of resulting LS samples after 90 days storage at 4°C.

To investigate the relationship between independent variables and their resulting response on the produced LS, 3D contour plots were constructed between them. In fact, from three independent variables, one was kept constant in each prepared plot, and the other two were changed. Figure 1 shows the first 3D constructed plot to show the relation of PVs with three levels of each ingredient. As this figure confirmed, the contents of RLP and HPMP had more positive effects than PGM on increasing PV. However, the PVs of LS samples prepared with different formulations were much less than 10 meq O_2_/kg even after 3‐month storage at 4°C. This value of PV has been used as a permissible level of fat oxidation in various food products because it is the start of fat alteration (Yadav et al., 2011).

**FIGURE 2:**
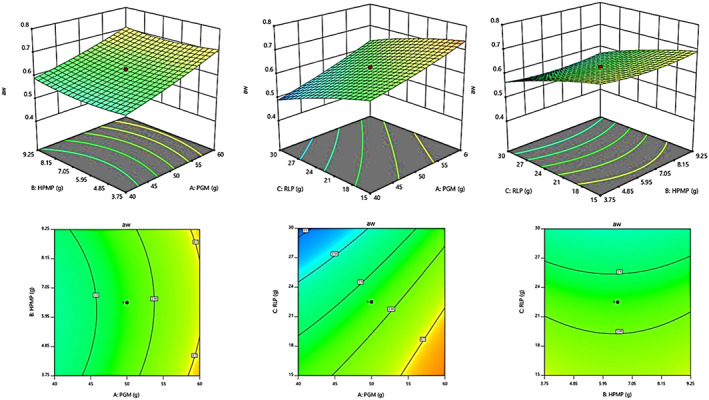
Response surface plots showing the interaction effects of independent variables (PGM, HPMP, and RLP) on the a_w_ of LS samples right after production.

Likewise, the results of Figure 2 confirmed that the contents of PGM and HPMP had more impacts than RLP on increasing a_w_ in the final linseed spread.

**FIGURE 3:**
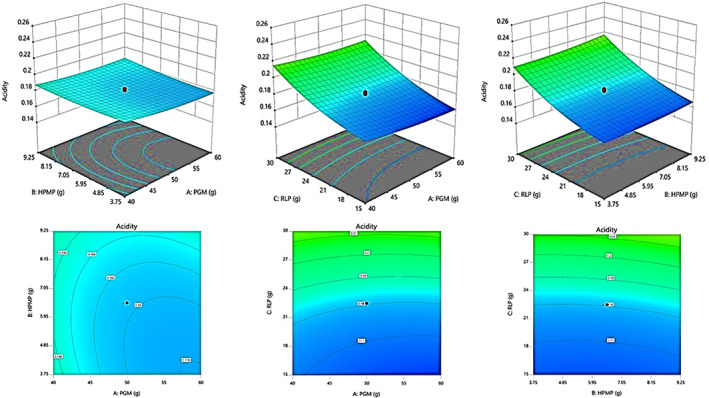
Response surface plots showing the interaction effects of independent variables (PGM, HPMP, and RLP) on the acidity with different LS samples right after production.

Figure 3 shows the percentage effects of three variables on free fatty acid (as oleic acid/100 g) of the final LS. The plots of this figure show that the RLP and HPMP increased the acidity of linseed spread; however, PGM had a decreasing effect.

### 3.2 Sensory evaluation

When nutritive and edible ingredients are processed properly and combined with safety and health concerns, there is a good chance to obtain a food product with good consumers' responses. In fact, customer opinion is a practical quality‐level evaluation. This is the reason profiling a new food product by using a trained panel's judgment is necessary to certify correctness and reproducing it with uniform sensory attributes. Despite the growing popularity of high‐quality cocoa and chocolate products, literature is limited on the sensory attributes of different nuts and specifically linseed spreads. The total sensory score obtained in the final LS product changed from 48.3 to 92.0 and it was related mainly to ingredients (PGM, HPMP, and RLP) proportion. As displayed in Table 2, only treatments 2, 7, 9, 10, 11, 12, 15, 16, 17, 19, and 20 obtained the acceptable acceptance sensory score (ASS) of more than 80% of the maximum ASS. However, the optimized LS (identified by using RSM) had 50 g PGM, 6.5 g HPMP, and 22.5 g RLP (treatment 19 of Table 2). Since the ASS of optimized SL was 110 out of maximum 120, the optimized SL could get up to 92% of the maximum score.

**TABLE 2:** The level effects of major ingredients on peroxide value, aw, acidity, and total sensory scores of LS (linseed spread) out of maximum samples along with central composite design arrangement right after production.

No.	Coded values			Actual values			Peroxide value (meq O_2_/kg)	Aw	Acidity (%OA)*	Acceptance sensory score (ASS)
	PGM	HPMP	RLP	PGM (g)	HPMP (g)	RLP (g)				
1	−1	−1	+1	40	3.75	30	1.38	0.50	0.216	71.4
2	+1	+1	+1	60	9.25	30	1.30	0.65	0.22	82.3
3	−1	+1	+1	40	9.25	30	1.40^a^	0.51	0.22	69.5
4	+1	−1	−1	60	3.75	15	0.74	0.76	0.159	72.1
5	−1	+1	−14	40	9.25	15	0.97	0.65	0.172	63
6	+1	−1	+1	60	3.75	30	1.21	0.65	0.209	75.8
7	0	0	0	50	6.5	22.5	1.02	0.63	0.178	89.2
8	0	0	−2	50	6.5	7.5	0.87	0.71	0.165	48.3
9**	0	0	0	**50**	**6.5**	**22.5**	**1.02**	**0.62**	**0.182**	**92**
10	0	0	0	50	6.5	22.5	1.04	0.63	0.183	87.5
11	0	0	0	50	6.5	22.5	1.03	0.63	0.18	89.2
12	0	−2	0	50	1	22.5	1.01	0.68	0.184	82
13	−2	0	0	30	6.5	22.5	1.12	0.53	0.195	51.8
14	−1	−1	−1	40	3.75	15	0.94	0.64	0.174	60.2
15	0	0	0	50	6.5	22.5	1.03	0.62	0.181	89.1
16	0	+2	0	50	12	22.5	1.12	0.69	0.192	87.8
17	+1	+1	−1	60	9.25	15	0.85	0.75	0.165	84.1
18	0	0	+2	50	6.5	37.5	1.78	0.46	0.258	59.3
19	0	0	0	50	6.5	22.5	1.03	0.62	0.18	91.2
20	+2	0	0	70	6.5	22.5	0.85	0.78	0.187	79.5

Note: *Oleic acid, **Best LS sample with the highest sensory score obtained by using CDC and RSM and choosing the optimized levels of GMP, HPMP, and RLP considering one level lower and one level upper for each ingredient.

### 3.3 Model fitting

Table 2 shows the level effects of three ingredients (PGM, HPMP, and RLP) on the chemical indexes (PV, a_w_, acidity) and sensory score of produced LS samples.

The linear and quadratic effects of RLP, PGM, and HPMP on dependent variables (PV, aw, acidity, and sensory score) along with checkup for their lack of fitness are explained in Table 3. The analyses of variance were also performed to determine the significance of independent variables on linear (first order) performance, their interaction effects, and their quadratic (second order) impacts on specifications of resulting LS.

**TABLE 3:** Regression coefficients and ANOVA of quadratic model for linseed spread.

Factor	Responses			
	Peroxide value (meq O_2_/kg)	Water activity a_w_	Acidity (% OA)	Acceptance (score)
Intercept	1.03	0.6252	0.1805	89.48
PGM	−0.0706	0.0631	−0.0028	6.60
HPMP	0.0294	0.0019	0.0022	1.94
RLP	0.2256	−0.0619	0.0238	2.60
(PGM) (HPMP)	0.0188	−0.0038	0.0019	2.20
(PGM) (RLP)	0.0063	0.0087	0.0019	−1.97
(HPMP) (RLP)	−0.0037	0.0012	0.0014	−1.27
(PGM)^2^	−0.011	0.0076	0.0025	−6.12
(HPMP) ^2^	0.009	0.0151	0.0017	−1.27
(RLP) ^2^	0.074	−0.0099	0.0076	−9.09
Model *F*‐value	2033.84 (Sig.)	192.89 (Sig.)	811.21 (Sig.)	153.47 (Sig.)
Lack of Fit *F*‐value	1.06 (Not Sig.)	0.4069 (Not Sig.)	0.2538 (Not Sig.)	0.9326 (Not Sig.)
*R* ^2^	0.9995	0.9986	0.9967	0.9928
Adj *R* ^2^	0.999	0.9974	0.9938	0.9863
Pre‐R2	0.9973	0.9963	0.9828	0.9666
Adequate Precision	190.6144	104.3536	73.3292	36.7261

The regression results showed that different prediction models tested for the four (PV, aw, acidity, and acceptance scores) response variables were highly adequate because they had satisfactory levels of *R* ^2^ (>99%) with no significant lack of fitness (Table 3). The empirical models for each response variable were developed by fitting its experimental data obtained from CCD design into a second‐order polynomial mathematical equation explained in Equation 1. As a result, four following predicted models with second‐order polynomial relationships were obtained for them (Equations (7), (8), (9), (10)):

(7) $$\begin{array}{cc} Y_{\text{Peroxide value}}= & +1.3639-0.002347\left(\mathrm{PGM}\right)-0.03475\left(\text{HPMP}\right)\\ & -0.032083\left(\mathrm{RLP}\right)+0.000682\left(\mathrm{PGM}\right)\left(\text{HPMP}\right)\\ & +0.000083\left(\mathrm{PGM}\right)\left(\mathrm{RLP}\right)-0.00011\left(\mathrm{PGM}\right)2\\ & +0.001187\left(\text{HPMP}\right)2+0.001315\left(\mathrm{RLP}\right)2 \end{array}$$

(8) $$\begin{array}{cc} Y_{\mathrm{aw}}= & +0.772391-0.00003040\left(\mathrm{PGM}\right)-0.006568\left(\mathrm{RLP}\right)\\ & +0.000233\left(\mathrm{PGM}\right)\left(\mathrm{RLP}\right)+0.000485\left(\text{HPMP}\right)\left(\mathrm{RLP}\right)\\ & +0.000084{\left(\mathrm{PGM}\right)}^{2}+0.001608{\left(\text{HPMP}\right)}^{2}-0.000095{\left(\mathrm{RLP}\right)}^{2} \end{array}$$

(9) $$\begin{array}{cc} Y_{\text{acidity}}= & +0.31889-0.003787\left(\mathrm{PGM}\right)-0.007122\left(\text{HPMP}\right)\\ & -0.004608\left(\mathrm{RLP}\right)+0.000068\left(\mathrm{PGM}\right)\left(\text{HPMP}\right)+0.000025\left(\mathrm{PGM}\right)\left(\mathrm{RLP}\right)\\ & +0.000025{\left(\mathrm{PGM}\right)}^{2}+0.000231{\left(\text{HPMP}\right)}^{2}-0.000136{\left(\mathrm{RLP}\right)}^{2} \end{array}$$

(10) $$\begin{array}{cc} Y_{\text{Acceptance}}= & -210.72108+6.85523\left(\mathrm{PGM}\right)+0.347746\left(\text{HPMP}\right)+9.33333\left(\mathrm{RLP}\right)\\ & +0.08\left(\mathrm{PGM}\right)\left(\text{HPMP}\right)-0.026333\left(\mathrm{PGM}\right)\left(\mathrm{RLP}\right)-0.061818\left(\text{HPMP}\right)\left(\mathrm{RLP}\right)\\ & -0.061227{\left(\mathrm{PGM}\right)}^{2}-0.173253{\left(\text{HPMP}\right)}^{2}-0.161515{\left(\mathrm{RLP}\right)}^{2} \end{array}$$

While raising levels of RLP and HPMP increased the peroxide value, raising PGM had decreasing impacts on the PV of the resulting LS (Equation 7) and Table 3). Similarly, the intercept values of a_w_ model (Equation 8) confirmed that the PGM and HPMP had increasing effects; however, the RLP had decreasing impact on a_w_ (Table 3).

Furthermore, (Equation 7) shows that (RLP)^2^ and (HPMP)^2^ with positive signs had more influences than (PGM)^2^ with a negative sign on increasing peroxide value. Likewise, Equation 8 shows that (HPMP)^2^ and (PGM)^2^ with positive signs had more impacts than (RLP)^2^ on increasing a_w_. Moreover, Equation 9 shows that all three (PGM, HPMP, and RLP) ingredients had significant impacts on the acidity of the final LS.

### 3.4 Optimization of the levels of independent and dependent variables for long storage

**FIGURE 4:**
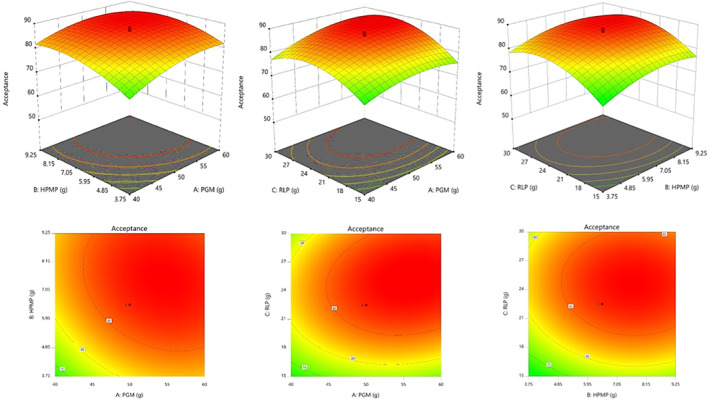
Response surface plots showing the interaction effects of independent variables (PGM, HPMP, and RLP) on the sensory acceptance score of LS.

Equation 10 shows that the three ingredients (PGM, HPMP, and RLP) had different influences on ASS of the final LS. Moreover, increasing the levels of the three major ingredients only to some extent improved the sensory evaluation score of LS (Figure 4), because the negative taste of RLP was noticed when it was increased by >27 g in the formulation. In fact, the higher content of RLP in the final LS prepared more unsaturated fatty acids (ready for oxidation), and therefore, more rancid fat (a kind of bitterness) taste was detected.

This is the reason that RSM use the ASS of different LS samples and found the optimum levels of each ingredient. Table 2 shows how this software suggested the best levels of three components for making optimized LS with the highest sensory attributes. The scale of desirability function used for this purpose ranged from 0 (completely undesirable response) to 1 (fully desired response), and it was corresponding to the meaningful quality levels including: very good (1.0–0.8); good (0.8–0.63); fair (0.63–0.37); poor (0.37–0.2); and very poor (0.2–0). The preferred goal for the desirable product was set within the ranges used for each independent variable.

**FIGURE 5:**
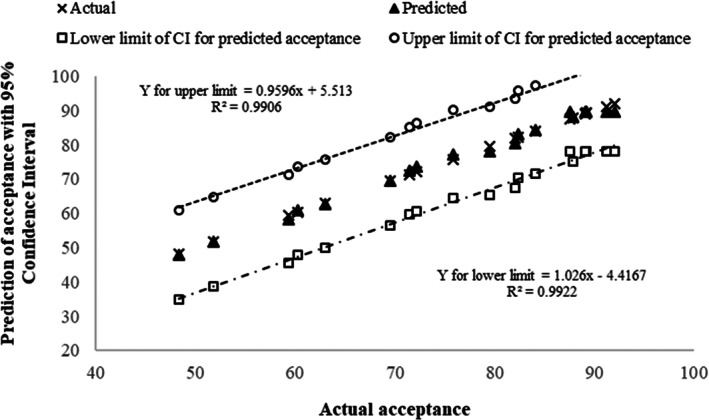
The predicted sensory scores of linseed spread (obtained from a regression model) versus its actual scores along with 95% upper and lower confidence intervals.

When the appropriate levels of RLP (22.5 g), PGM (50 g), and HPMP (6.5) were substituted, respectively, in mathematical models of PV, w_a_, acidity, and ASS (Equations of 7, 8, 9, and 10); the absolute differences (or nonconformities) between the actual values of PV, a_w_, acidity, and sensory score with their theoretical (or predicted) values were <4% (see Table 4). This means that the suggested proportion of the three major ingredients had good potential to produce attractive linseed spread for consumers. Furthermore, Figure 5 clearly shows the high consistency of the predicted sensory scores with those obtained in the actual experiment.

**TABLE 4:** The practical ranges and appropriate levels of three independent variables for making LS with highest sensory score and permissible chemical indexes (PV, a_w_, and acidity) along with comparison between the predicted and actual sensory scores.

	Acceptable ranges	Experimental values for optimized LS sample	Predicted value	Error (%)
PGM (g)	40–60	50	48.053^a^	3.89
HPMP (g)	3.75–9.25	6.5	6.277^a^	4.20
RLP (g)	15–30	22.5	21.455^a^	4.64
Peroxide value (meq O_2_/kg)	0.74–1.78	1.02	1.023^b^	0.29
aw	0.54–0.88	0.70	0.682^b^	2.57
Acidity (% oleic acid)	0.159–258	0.182	0.179^b^	1.64
Acceptance (sensory score)	50–88.3	88.3	84.893^b^	3.85

^a^ The theoretical values of PGM, HPMP, and RLP numbers were gained when the actual PV, aw, acidity, and acceptance sensory score (obtained by experiment) were inserted in equations of 7, 8, 9, and 10.

^b^ The three chemical indexes and acceptance sensory score were obtained when the appropriate levels of PGM, HPMP, and RLP were inserted in equations of 7, 8, 9, and 10.

Besides the discussed dependent variables (PV, aw, and acidity), other chemical parameters of LS including the fiber, protein, fat, and ash contents along with pH had an influence on the sensory attributes and textural behavior of the final product. Table 5 shows that the PV and acidity of the optimized sample of LS with ~11% (mainly linseed oil) increased, respectively, from 1.02 to 1.70 meq O_2_/kg and 0.182 to 0.199% after 90‐day storage at 4°C. However, the PV and acidity of hazelnut–cocoa spread stored for 90 days at similar conditions were much higher and, respectively, changed from 4 to 6 meq O_2_/kg and 0.71 to 0.77% mainly because it had more fat (10% palm oil and 11% sunflower oil) with lower quality (Tarakçi & Yildirim, 2021). Although the protein content of some nut spreads (such as peanut spread) is >20% (Mazaheri‐Tehrani et al., 2009) and higher than LS, our suggestive spread has considerable and unique health benefits mainly attributed to its omega‐3 fatty acids, fiber, and lignin (Heuzé et al., 2015). Additionally, 3‐month storage of LS did not make significant changes to its protein (~7%), fat (~11%), and ash (~1%). Nevertheless, its acidity and PV values were below the permissible levels for food products even after a 90 days storage at 4°C.

**TABLE 5:** Chemical properties of optimized linseed spread.

Storage time	Factor							
	Fiber (%)	Protein (%)	Fat (%)	Ash (%)	pH	Aw	Acidity (%OA)	Peroxide value (meq O_2_/kg)
1st day	5.17 ± 0.35^a^	12.45 ± 1.14^a^	9.52 ± 1.28^a^	2.32 ± 0.92^a^	6.12 ± 0.12^a^	0.62 ± 0.09^a^	0.182 ± 0.03^d^	1.02 ± 0.12^c^
30th day	5.12 ± 0.22^a^	12.42 ± 1.21^a^	9.50 ± 1.47^a^	2.27 ± 0.87^a^	5.72 ± 0.18^b^	0.61 ± 0.08^a^	0.190 ± 0.02^c^	1.12 ± 0.10^b^
60th day	5.15 ± 0.28^a^	12.38 ± 1.12^a^	9.32 ± 1.33^a^	2.31 ± 1.03^a^	5.58 ± 0.21^c^	0.58 ± 0.11^a^	0.196 ± 0.01^b^	1.22 ± 0.14^a^
90th day	5.14 ± 0.43^a^	12.43 ± 1.19^a^	9.55 ± 1.25^a^	2.34 ± 0.69^a^	5.39 ± 0.15^cd^	0.56 ± 0.09^a^	0.199 ± 0.02^a^	1.50 ± 0.17^a^

*Note*: *For each column, means ± SD with different (a, b, c, and d) superscripts are significantly different (*p* < .05).

### 3.5 Physical properties (color values and texture analysis)

In terms of customer thought and quality perception, the appealing features of hazelnut and chocolate spreads are crucial. Consumers are interested to accept spread products with a smooth and shiny surface. While the linseed spread does not generate fat blooms appearance (which is a significant problem in chocolate products), its color values (L, a, and b) had important roles in making smooth surface color. The L value of LS was very close to the chocolate spread (~30); however, its a (redness to greenness) and b (yellowness to blueness) values, respectively, were higher and lower than the chocolate spread. Researchers reported that the L, a, and b values of the chocolate spread are ~30, 12, and 22, respectively (Almeida & Lannes, 2017). Most probably, a higher value of LS was due to the usage of Persian grape molasses, which has a dark red color.

**FIGURE 6:**
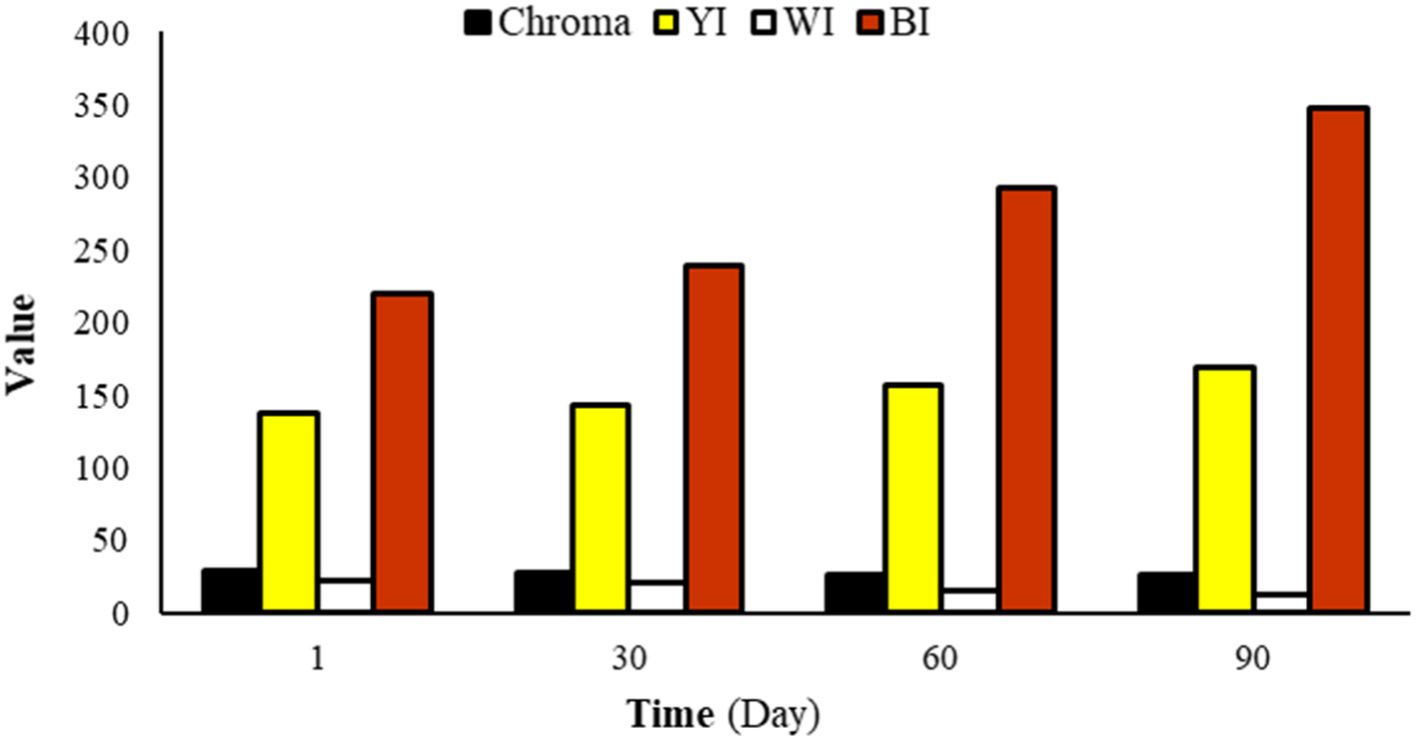
Effect of storage time on color pigment (WI, YI, BI, and Chroma) of optimized linseed spread at 4°C storage.

Figure 6 shows the changes of color parameters including chroma, whitish (WI), yellowness (YI), and brownness (BI) indexes of the LS samples during 90 days storage at 4°C. While its BI and YI parameters were increased during the days of storage (due to the browning and oxidation processes), its Chroma and WI reduced and made a product with a darker color; however, their changes in chroma, WI, and YI during cold storage were not significant. Researchers believe some complicated chemical processes involving sugars, acids, and phenolic compounds happen during storage of each nut and chocolate spread even at 1–5°C, and these reactions may reduce their astringency and bitterness, resulting in a more stable color, pleasant smell, and superior flavor in different (nuts and chocolate) spreads (Baptista et al., 2021; Lončarević et al., 2018). Storage conditions (mainly temperature and relative humidity) significantly altered the scales of color values and parameters (Andrae‐Nightingale et al., 2011). While the storage of dark and milk chocolate in various conditions for 5 weeks reduces their visual and textural quality mainly because of sugar or fat bloom formation (Andrae‐Nightingale et al., 2009), the chroma, WI, and YI of suggestive linseed spread were not involved with degradation and defects during storage.

**FIGURE 7:**
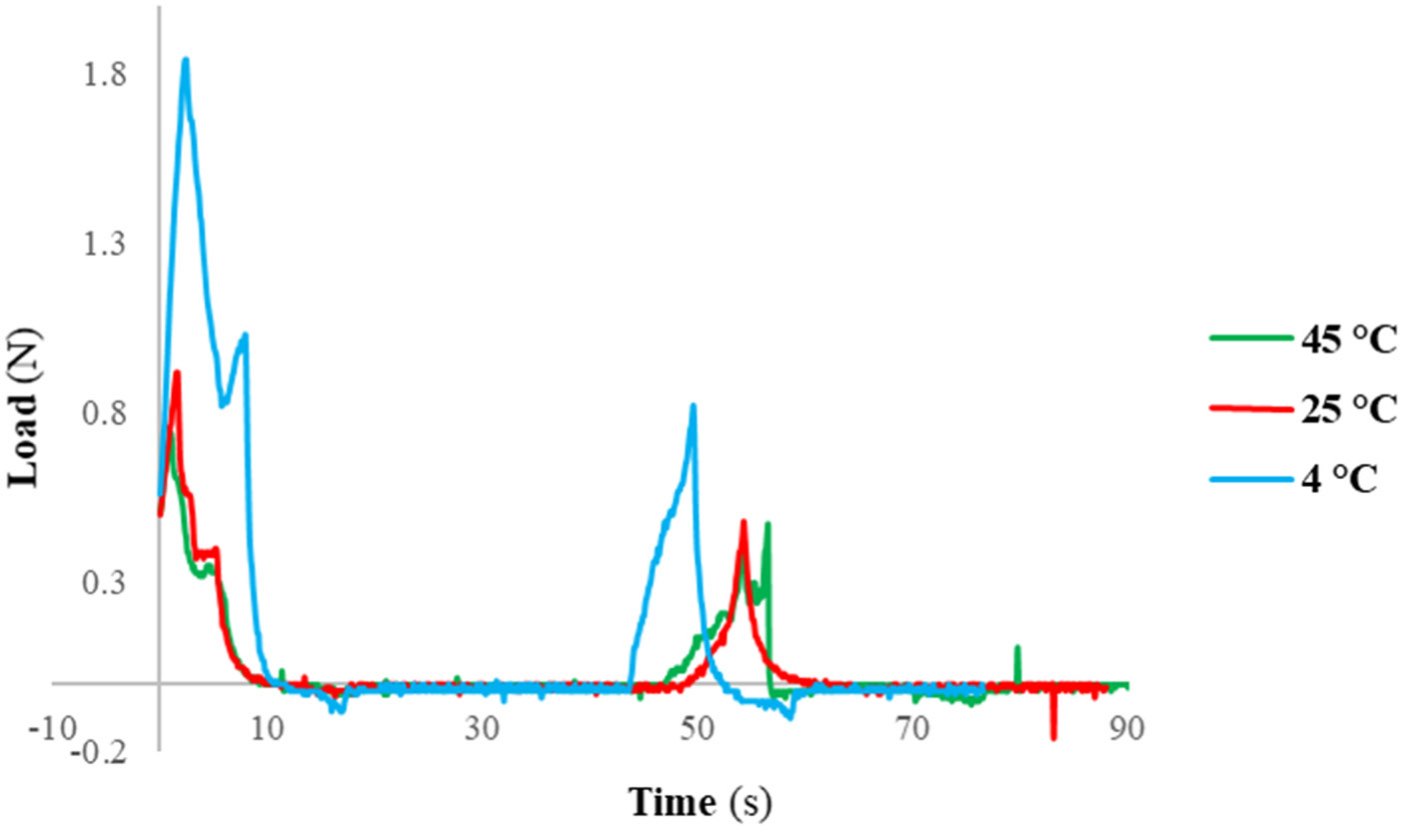
Effect of storing temperature on the hardness (the peak forces measured for the first compression cycle) of optimized linseed spread after its production.

Figure 7 and Table 6 show the behavior profile of linseed spread after a double compression test. The analysis showed that the hardness (the peak force measured in 10 s in the first compression cycle) of optimized LS at three temperatures of 4, 25, and 45°C was 1.84, 0.92, and 0.74 N, respectively. The adhesiveness or the negative force area (which happened after the first bite of LS within 15 s) was very small (≤ − 0.2 N) for each sample. In fact, this force was necessary to pull the compression probe away from each sample. The gumminess, which is related to the force needed to disintegrate each sample of LS in the mouth (until it is ready to swallow) at 4°C was equal to 0.6 N, and it was much higher than the gumminess of LS at 25 and 45°C.

**TABLE 6:** Texture parameters of optimized linseed spread on first day of production.

Temperature	Hardness (*N*)	Adhesiveness (mJ)	Cohesiveness	Springiness	Gumminess (*N*)	Chewiness (*N*)
4°C	1.84	0.4	0.33	0.26	0.60	0.16
25°C	0.92	0.3	0.32	0.24	0.27	0.06
45°C	0.74	0.2	0.29	0.21	0.25	0.05

The springiness measures a force needed to return the LS product to its original size/shape after partial compression (without failure). This force is applied between the tongue and teeth (palate) for reaching to a suitable consistency. It means that the food with higher elasticity needs higher grinding energy. In contrast, chewiness measures the time it takes to chew a sample until it reaches a consistency that is suitable for swallowing. Although the springiness force of the LS sample at 4°C was ~0.26 N, it was again significantly more than those for 25 and 45°C. However, its chewiness was considerably lower (easier) than the similar one at a temperature > 25°C. This may be due to the reverse effects of temperature and the presence of RLP, which both increase the viscosity. On the other hand, mastication is a complicated process that involves a variety of mechanical forces, and it is difficult to measure chewiness accurately. In contrast with chocolate spread, where the inclusion of corn syrup and butter increased its viscosity and adhesiveness (Dagadkhair et al., 2018; Karavasili et al., 2020), the optimized LS was low sticky (0.2–0.4 mJ = 2‐4x10^−4^ N‐m). Stickiness means the force needed to adhere the product to the lips for mouth feel.

Storage condition significantly impacted instrumental hardness, cohesiveness, adhesiveness, springiness, gumminess, and chewiness.

### 3.6 Rheological description

The storage modulus (G') shows the elastic response (or stored energy) of food and reflects its solid‐state behavior. However, loss modulus (G") reveals the viscous response (or dissipated heat energy) of food (such as spread) and shows its liquid‐state behavior (Mantihal et al., 2019). Furthermore, internal friction between the components (molecules and particles) of a moving fluid causes viscous behavior and the production of frictional heat. This friction is always associated with the energy conversion of deformation to heat dissipation. When the food material is deformed, the unused stored energy is a driving force to reconstruct into its original shape (Pajin et al., 2013).

**FIGURE 8:**
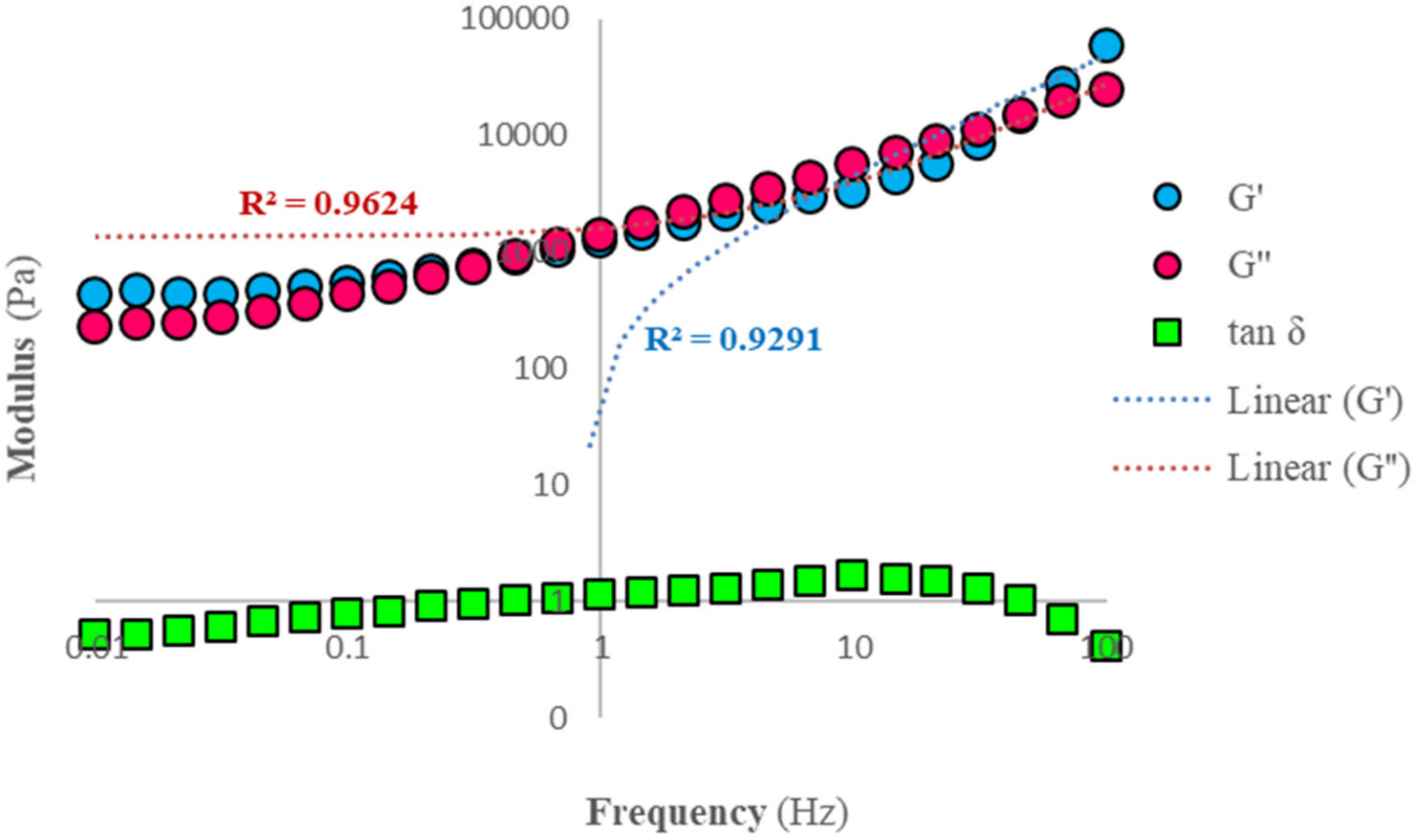
The effects of frequency (applied forces) on storage modulus (G'), loss (G'‘) modulus, and tan δ at 25°C in the linear viscoelasticity region of linseed spread. The units for both moduli are pressure or energy/unit volume of LS after production.

Figure 8 illustrates the development of the storage (G') and loss (G") moduli because of linear range of viscoelasticity of LS versus applied frequency. At frequencies below 1 Hz, the storage modulus values were considerably more than those in loss modulus (G' > G”). This is owing to the internal (chemical bonds connections, physiochemical) interactions between the molecules and particles of the materials in the resulting spread (Janmey & Schliwa, 2008). However, the situation of these two moduli was reversed when the frequency became higher than 1 Hz, and subsequently, the loss modulus rose more than the storage modulus.

Figure 7 also shows the loss tangent values (tan δ = G”/G') as a function of frequency. This is also known as the gel point or the sol/gel transition point and indicates that the sample moved from a liquid (or sol state) to a solid (or gel state) and vice versa during the viscoelastic measurement at different frequencies. Researchers reported that the optimized linseed spread behaves like a viscous material when the tan δ > 1. On the other hand, when tan δ < 1, it behaves like an elastic matter (Manasi, 2019; Qaiser et al., 2021). These findings can be explained by the fact that the RLP plays a dual role in the formulation of LS and acts as a filler in the complicated network of the resulting spread. In other words, significant interactions take place between crystallized HPMP and PGM, and their outcomes make hydrodynamic polymers with the oil base of linseed paste. Researchers believe that the low total elasticity of spreads is related to their high particle concentrations (Taylor et al., 2009).

Lecithin has been used in nut (and specifically chocolate) spreads to control their viscoelasticity in the confectionery industry. This is the reason that cocoa butter has been replaced with lecithin because lecithin has strong emulsification power and is much less expensive than cocoa butter. Moreover, the usage of soy lecithin at 0.1–0.3% level in every spread can reduce the same viscosity as over 10 times this amount of cocoa butter (Karnjanolarn & McCarthy, 2006). However, if the level of lecithin exceeds 0.5%, unsuitable thickening of the chocolate occurs (Karnjanolarn & McCarthy, 2006). This is the reason we used lecithin below 0.5% to control the rheology of the final LS.

The optimized LS had viscoelastic properties because RLP plays a dual role and acts as a filler in its complicated network.

Most probably significant interactions take place between crystallized HPMP and PGM to make hydrodynamic polymers with the oil base of linseed paste. While the linseed spread had high protein content, considerable unsaturated fatty acids, and antioxidants, it showed a long shelf life at 4°C storage. Additionally, it could predict the ASS of new LS without any sensory test for reassessment.

## CONCLUSION

The optimized LS (linseed spread) produced with appropriate levels of roasted linseed paste, Persian grape molasses, and high protein milk powder had high consumer acceptability. The fine particles of optimized LS made a tasty cream and nutritive food for children due to its high protein, unsaturated fatty acids, and antioxidants.

While its a_w_, acidity, and PV were very low during 90 days storage at 4°C, their changes even after this time were insignificant and below the permissible level. Furthermore, the optimized LS had acceptable color, textural, and viscoelastic properties.

Since the mathematical model prepared for sensory scores had a high consistency with those obtained in the actual experiment, this model can be used to predict the sensory score of LS sample with similar formulation without having to impanel a new set of tasters to reassess its sensory attributes. The optimized LS had high nutritional values (high protein, unsaturated fatty acid, and fiber) and has a good potential to be accepted in the international markets due to its high sensory attributes and nutritional aspects.
